# Using Machine Learning to Predict Uptake to an Online Self‐Guided Intervention for Stress During the COVID‐19 Pandemic

**DOI:** 10.1002/smi.70032

**Published:** 2025-04-22

**Authors:** Gavin N. Rackoff, Michelle G. Newman

**Affiliations:** ^1^ Department of Psychology The Pennsylvania State University University Park Pennsylvania USA

**Keywords:** digital health, dissemination, healthcare utilization, implementation, machine learning

## Abstract

Online self‐guided interventions appear efficacious for alleviating some mental health concerns. However, among persons who are offered online interventions, only a fraction access them (i.e., achieve uptake). Machine learning methods may be useful to predict who will achieve uptake, which could inform improvements to interventions and their methods of delivery. We used secondary data from participants given access to a self‐guided online stress intervention during the COVID‐19 pandemic in a randomised trial (*N* = 301, among whom 158 achieved uptake). This study built and evaluated several models for predicting uptake. Putative predictors included demographic characteristics, mental health service utilization and interest, and mental health symptoms assessed before participants were provided access to the intervention. The best‐performing model, a linear support vector machine model, had 70% accuracy and 0.70 area under the receiver operating characteristics curve in a held‐out dataset, though these metrics were not significantly better than competitor models. Model inspection revealed that participants who reported interest in mental health treatment and lesbian, gay, bisexual, and other sexual minority participants were more likely to achieve uptake. Additionally, male participants were less likely to achieve uptake. The best‐performing machine learning model achieved an acceptable level of performance in predicting uptake. Self‐reported treatment interest was especially predictive of uptake. Future research should attempt to understand gender and sexual orientation differences in self‐guided online mental health intervention uptake. Additionally, research should evaluate the utility of machine learning to inform targeted motivational enhancement of those less likely to achieve uptake.

## 1 Introduction

The past 2 decades have seen a boom in the availability of online mental health interventions (Torous et al. 2019). Given the widespread use of computers and smartphones (Pew Research Center 2021) and limited supply of traditional mental healthcare providers such as psychologists and psychiatrists (Thomas et al. 2009), online interventions have potential to vastly increase access to mental health support. Moreover, many online interventions are fully self‐guided, allowing users to proceed through interventions without the involvement of a human provider and further increasing scalability. Trials have suggested that self‐guided online interventions can be efficacious for alleviating common mental health concerns such as depression and anxiety (Edge et al. 2023; Karyotaki et al. 2017). Nonetheless, a major challenge facing online self‐guided interventions is limited usage. Illustrating this challenge, meta‐analyses comparing self‐guided online interventions to coach‐ or therapist‐guided interventions have found lower usage (Musiat et al. 2022) and smaller treatment effects (Moshe et al. 2021) from self‐guided interventions. Understanding and improving usage of self‐guided digital interventions is crucial to realise their potential.

A basic yet pivotal aspect of usage is uptake, defined as whether or not a person accesses an intervention at least once after being offered the intervention. Uptake is a key bottleneck given that, no matter how efficacious an intervention is, if a person never accesses it, that person cannot benefit directly from it at all. Moreover, whereas other metrics of usage could vary in their importance across digital interventions (e.g., interventions designed to be used for different amounts of time and different frequencies of use), uptake is applicable across interventions as the first step in treatment. Relatedly, the interpretability and importance of uptake remains consistent even if a digital intervention provider updates its service over time, such as by adding or removing content. Uptake therefore represents a practically important phenomenon due to its influence on subsequent intervention delivery and its applicability across digital health services.

Trials of online mental health interventions have yielded a wide range of uptake rates, which have been summarised in several systematic reviews. A meta‐analytic review of online cognitive‐behavioural intervention studies targeting college students (D'Adamo et al. 2023) found that uptake averaged 79% across studies, with a range of 32%–100%. Another meta‐analysis examined uptake of smartphone‐delivered interventions for a range of mental health concerns (Linardon and Fuller‐Tyszkiewicz 2020). Among the reviewed studies with uptake data, most (eight studies) targeted general mental health as opposed to a specific symptom such as depression or insomnia, and across these studies, the average rate of uptake was also 79%, with a range of 57%–100%. Fleming et al. (2018) reviewed studies of self‐guided online interventions for emotional problems. Rates of at least minimal use (e.g., logging into the intervention platform at least once) ranged from 21% to 84%, and averaged 56%. Unlike the other two reviews, Fleming et al. (2018) explicitly focused on self‐guided and ‘real‐world’ digital intervention studies. Hence, they excluded randomised trials, potentially explaining the lower average rate of uptake. Thus, the range of reported rates of uptake into online intervention studies is wide, and especially when considering fully self‐guided interventions, a very high proportion of potential users do not receive any intervention dose. The suboptimal and variable rates of uptake into online programs is likely to limit their impact.

Machine learning methods have potential to help the field understand and improve uptake into online self‐guided interventions. As defined by Dwyer et al. (2018), machine learning is ‘a computational strategy that automatically determines (i.e., learns) methods and parameters to reach an optimal solution to a problem’ (p. 94). Using such a model, it could be possible to triage individuals to an intervention who are likely to achieve uptake, while referring those not likely to achieve uptake to receive extra messaging or incentives first. For example, some evidence has suggested that motivational interviewing (Dean et al. 2016) or interactive text message‐based reminders about available services (Fitzsimmons‐Craft et al. 2024) could increase the probability of mental health services uptake. Thus, machine learning could help identify persons unlikely to achieve uptake and target them with supplementary interventions meant to increase uptake. Alternatively, individuals with a low probability of uptake could be triaged to receive a different kind of intervention, although treatment settings will vary regarding which (if any) alternative interventions are available. Machine learning models can also aid in theory building. For example, a predictive model could provide insights about the kinds of people who do versus do not achieve uptake. These insights could illustrate sectors of the population for whom a given intervention is not appealing or for whom different strategies are needed. Thus, machine learning can be used to optimize online intervention development and dissemination.

Machine learning model performance can be evaluated using various metrics. These metrics include sensitivity (the percentage of uptake outcomes correctly predicted), specificity (the percentage of non‐uptake outcomes correctly predicted), accuracy (the percentage of correctly classified outcomes), and area under the receiver operating characteristics curve (AUC). Best practices suggest that models should be built and evaluated in a ‘training’ dataset and then subsequently evaluated in a separate ‘testing’ dataset (James et al. 2021). This separation is employed to ensure that model performance is not overestimated in the event that it is overfit, or overly swayed by random noise in the training dataset. In addition to evaluating performance, it is informative to examine model parameters, or in other words, estimated relationships between the predictors and the outcome (e.g., uptake).

A growing literature has used machine learning to predict digital mental health intervention uptake. In a combined sample from three trials of four‐session, self‐guided cognitive‐behavioural online eating disorder interventions (*N* = 826), Linardon et al. (2022) found that the best‐performing model had 100% sensitivity and 0% specificity, with a chance‐level AUC of 0.50 in the testing dataset. The authors concluded that the machine learning models performed poorly. In a large sample (*N* = 15,882) of Australian adults (Cross et al. 2022), uptake was predicted for a digital mental health platform providing several eight‐week programs for a range of mental health concerns (e.g., general well‐being, obsessive‐compulsive disorder, posttraumatic stress), and with the availability of therapist consultation (as described by Titov et al. 2020). The machine learning model achieved 67% sensitivity, 57% specificity, and an AUC of 0.66, though no training‐testing split was described. Variables associated with a higher probability of uptake were older age, lower distress severity, absence of drug difficulties, being male (vs. female), and not being a student. Finally, Vera Cruz et al. (2023) used machine learning to predict self‐reported use of any smartphone applications for any type of mental health or general well‐being in a survey of 1989 U.S. community‐dwelling adults. This model achieved an accuracy of 71%. Variables associated with greater probability of uptake included younger age, being female (vs. male), absence of substance abuse problems, and presence of self‐reported emotional problems.

Taken together, previous studies using machine learning to predict online intervention uptake have both affirmed the promise of these methodologies and highlighted the need for further research to clarify their utility and identify consistent predictors of uptake. Two of the three prior studies (Cross et al. 2022; Vera Cruz et al. 2023) suggested that machine learning models could accurately predict online mental health intervention uptake at better than chance levels. However, only Vera Cruz et al. (2023) described a rigorous train‐test split for model evaluation. Nonetheless, this study examined self‐reported digital health service use, rather than objective uptake among participants offered a service, the latter of which is likely to be more generalisable to real‐world implementation settings. Additionally, prior studies yielded divergent substantive findings on how uptake was related to age, emotional distress, and gender, though they converged in suggesting that substance use problems were associated with a lower probability of uptake. Further investigation of the potential accuracy of machine learning models and predictors of uptake is necessary to justify real‐world implementation of these methodologies. Notably, real‐world implementation settings might not allow assessing a wide range of putative predictors of uptake as in randomised trials, and tailored intervention delivery could therefore benefit from knowledge of a small subset of easily‐assessed predictors of uptake. With this in mind, it would be valuable to incorporate self‐reported interest in receiving treatment as one of the predictors in machine learning models. Though not examined in prior machine learning studies, interest in receiving treatment can be assessed with as little as one question, and if it emerges as a strong predictor of uptake, it could be easily incorporated into predictive models and used to inform targeted intervention delivery (e.g., motivational interviewing phone calls prior to offering a mental health service to persons uninterested in treatment). The present research aimed to address the need for further rigorous investigation of the accuracy of machine learning methods for prediction of uptake and knowledge of easily assessed predictors of uptake.

Uptake is a major limiting factor to the impact of online self‐guided mental health interventions. Machine learning can be used to develop models to predict uptake, which may inform novel methods of intervention dissemination and delivery. Additional work using machine learning methods is needed to better understand their potential and provide substantive information about predictors of uptake. To address this need, this research had the following aims: (1) to develop a set of machine learning models to predict uptake into online mental health interventions, (2) to evaluate the accuracy of these models in predicting uptake and select a best‐performing model, and (3) to examine the implications of the best‐performing model to yield insights about possible determinants of uptake.

## 2 Method

### 2.1 Participants

This study used secondary data from a published trial of an online self‐guided intervention for stress related to the COVID‐19 pandemic (Rackoff et al. 2022). Participants included all *N* = 301 individuals who were randomly assigned to receive the self‐guided intervention (vs. usual care control). Among them, 158, or 52% achieved uptake. The trial specifically targeted the college population and therefore all participants were students enroled at a U.S. college or university. Participant characteristics are shown in Table 1.

**TABLE 1:** Participant characteristics.

Variable	*N*	%
Uptake		
Yes	158	52
No	143	48
Age		
Traditional college age (18–21)	265	88
Non‐traditional college age (22+)	36	12
Gender		
Female	216	72
Male	65	22
Other identity	20	7
Sexual orientation		
Heterosexual	188	62
LGB+	113	38
Racial/ethnic minority status		
White, non‐hispanic	214	71
Racial/ethnic minority or non‐disclosed	87	29
Year in school		
Undergraduate year 1	116	39
Undergraduate year 2	92	31
Undergraduate year 3	57	19
Undergraduate year 4	20	7
Other	16	5
School region		
Northeast	197	65
South	84	28
Midwest	10	3
West	10	3

*Note: N* = 301.

Abbreviation: LGB+ = lesbian, gay, bisexual, or other sexual minority identity.

### 2.2 Procedures

See Rackoff et al. (2022) for a full description of study procedures. In brief, eligible participants were identified via an online screening survey that assessed the following criteria (1) age 18 or older, (2) student at a U.S. university, (3) a score of 20 or higher on the Depression Anxiety Stress Scales‐Short Form, stress subscale (Antony et al. 1998). The screening survey was only sent to participants who were on a participant registry. Participants either opted in to the registry by responding to online advertisements or were included in the registry through the use of school administrator‐verified lists of students and associated email addresses. The survey was programed to prevent duplicate responses with personalised survey links and to prevent fraudulent responses with CAPTCHA. Eligible participants were immediately invited to participate and complete a baseline survey prior to inclusion. All participants provided informed consent for screening, the baseline survey, and the trial. Together, the screen and baseline survey assessed all predictors in the present study's analyses. Participants who completed the baseline survey were included in the trial and randomly assigned to a trial arm via 1:1 ratio. The screen, baseline surveys, and randomisation were administered via Qualtrics survey software.

Immediately upon random assignment to the intervention condition, the Qualtrics survey informed participants that they had been selected to receive access to the online intervention. The survey provided a brief description of the intervention and the two available programs. In brief, the two programs consisted of (1) a cognitive‐behavioural therapy‐informed program designed to deliver stress management skills for coping with the COVID‐19 pandemic and (2) a positive psychology program delivering resilience skills. Participants were told they could use either or both of the programs, and they could switch between programs over time; for more details about the programs, see Rackoff et al. (2022). The description of the intervention included hyperlinks to access the intervention. Participants who did not click one of the hyperlinks to access the intervention were emailed a reminder, which also included hyperlinks to access the intervention. These hyperlinks were personalised to each participant. Thus, uptake was defined as whether a participant clicked the link to begin the intervention, and uptake occurred prior to a participant's initial selection of a program. Participants initially selected to work on the positive psychology resilience program most commonly (*n* = 124, 78% of those with uptake), followed by the COVID‐19 stress management program (*n* = 19, 12% of those with uptake); an additional 15 participants (9% of those with uptake) accessed the intervention but did not select either program after accessing the platform. There was too little variability to build machine learning models predicting program selection, and descriptive analyses revealed no significant associations between participant characteristics and program selection behaviour among participants who achieved uptake (see Supporting Information S1); thus, all participants who accessed the intervention at least once, irrespective of program selection behaviour, were collectively considered to have achieved uptake, and uptake was the outcome in machine learning analyses. The description of the intervention shown in the survey and follow‐up email are also in the Supporting Information S1.

### 2.3 Measures

#### 2.3.1 Uptake

As noted above, uptake was defined as whether a participant accessed the intervention at least once by using the personalised link.

#### 2.3.2 Predictor Variables

The following demographic variables were assessed: age, gender, sexual orientation, racial/ethnic minority status, year in school, and school geographic region. Participants were asked yes‐or‐no questions about current mental health treatment (‘Are you currently receiving any treatment for psychological difficulties?’) and mental health treatment interest (‘Are you currently interested in seeking treatment for psychological difficulties?’). The survey also assessed a wide range of mental health symptoms using validated self‐report measures. The Depression, Anxiety, Stress Scales‐Short Form (DASS‐SF; Antony et al. 1998) assessed depression, anxiety, and stress across three subscales. The Perceived Stress Scale (PSS‐10; Cohen and Williamson 1988) assessed perceived stress. The Patient Health Questionnaire‐9 (PHQ‐9; Kroenke et al. 2001) assessed depression. The Generalised Anxiety Disorder Questionnaire‐IV (GAD‐Q‐IV; Newman et al. 2002) assessed generalised anxiety disorder symptoms. The Social Phobia Diagnostic Questionnaire (SPDQ; Newman et al. 2003) assessed social anxiety disorder symptoms. The Weight Concerns Scale (WCS; Killen et al. 1996) assessed concerns about weight and body image. The Alcohol Use Disorders Identification Test (AUDIT‐C; Bush et al. 1998) assessed alcohol abuse. The Insomnia Severity Index (ISI; Bastien et al. 2001) assessed insomnia. The Primary Care Posttraumatic Stress Disorder Screen (PC‐PTSD; Prins et al. 2003) assessed posttraumatic stress symptoms. Each measure was scored dimensionally, with a higher score indicating greater severity.

### 2.4 Data Analyses

#### 2.4.1 Data Preparation

Analyses in this manuscript were not pre‐registered. All data and analytic code can be found at https://osf.io/y7t54/?view_only=bb1d3277adff4572b6313636b29f14a2. Please see Supporting Information S1 for detailed information about data preparation. Stratified sampling was used to select a training dataset of 234 (78%) observations, with the remaining 67 observations reserved for the test dataset. Stratified sampling was implemented to ensure that the training dataset and testing dataset were both representative of the full sample. This avoided the problem known as ‘covariate shift’, in which covariates differ substantially across the train and test data (see López et al. 2014 for a discussion). There was no missing data on uptake, and minor missing data on predictors (*n* = 7, or 2% had missing data on at least one predictor) was handled using single regression imputation models as estimated in the training data. To reduce dimensionality relative to the small number of observations and handle collinearity among symptom variables, a principal components analysis (PCA) reduced symptoms to one dimension that explained 38% of their variance in the training data. This dimension can be understood to capture general distress; the only variable not substantially captured by this dimension (absolute loading < 0.1) was AUDIT‐C, which was retained as an additional predictor. A sensitivity analysis revealed that skipping the PCA step in data preparation and retaining each symptom as an individual predictor did not result in any models that outperformed the best‐performing from those trained after conducting PCA; thus, this alternative set of analyses is not reported in the main text but can be found in the Supporting Information S1.

#### 2.4.2 Predictive Modelling

We evaluated the performance of four modelling strategies: logistic regression (LR), support vector machine with linear kernel (SVM‐Linear), support vector machine with polynomial kernel (SVM‐Polynomial) and random forest (RF). Each method was tuned in the training data to optimize model performance. For LR, we used an elastic net penalty, which combines ridge (shrinkage) and LASSO (variable selection) penalties, and we tuned the parameters alpha, which controlled the balance of ridge versus lasso penalty, and lambda, which controlled the strength of the penalty. For SVM‐Linear, we tuned the parameter cost, which controlled the narrowness of the margin around the decision boundary. For SVM‐Polynomial, we tuned cost, degree of the polynomial, and gamma, which controlled the kernel function. RF tuning parameters included the number of variables considered within each tree split and the number of trees used in aggregate. For LR, SVM‐Polynomial and RF, which involved more than one tuning parameter, tuning parameters were selected using a grid search (i.e., evaluating every possible combination among a selected set of values for each parameter). Tuning was performed with six‐fold cross validation using the training data.^1^ Analyses were performed in *R* 4.1.1 (R Core Team 2021) with the *glmnet* package (Friedman et al. 2010; Tay et al. 2023) for LR, the *e1071* package (Meyer et al. 2023) for SVM‐Linear and SVM‐Polynomial, and the *randomForest* package (Liaw and Wiener 2002) for RF. After tuning the hyperparameters, we subsequently tuned the binary classification threshold by selecting the value that maximised Youden index reflecting the optimal balance between sensitivity and specificity. The threshold was tuned in the training dataset using the *pROC* package in *R* (Robin et al. 2011). Tuning grids and selected parameters are in Supporting Information S1.

For each trained model, we evaluated sensitivity, specificity, accuracy, and AUC in the training and testing datasets. AUC of 0.5 or lower is considered zero discrimination, 0.51–0.69 is considered poor, 0.7–0.79 is considered ‘acceptable’, 0.8–0.89 is considered ‘excellent’, and greater than or equal to 0.9 is considered ‘outstanding’ (Hosmer et al. 2013). The optimal model was the one with the most favourable performance in the testing dataset. Because AUC evaluates performance across all possible decision thresholds, it was used as the primary performance metric for model selection, with accuracy as a tie‐breaker. Accuracy, as a threshold‐dependent metric, was an appropriate tie‐breaker because it could indicate how well a model would perform when used to make a binary decision (e.g., whether or not to send a referred individual additional motivational reminders to promote uptake). Thus, selecting models with both AUC and accuracy represented a comprehensive approach to model evaluation. We compared models using both point estimates of AUC and accuracy as well as formal statistical tests. We conducted pairwise comparisons of AUC using the DeLong test and accuracy using the McNemar test. The null hypothesis for these tests stated that the models did not differ in AUC or accuracy, and hence a statistically significant result would indicate superior performance for a given model.

After selecting the optimal model, we examined model‐implied relationships between predictors and uptake. We used Shapley Additive Explanation (SHAP) values (Lundberg and Lee 2017) to interpret the selected model. SHAP values provided estimates of the influence of a given participant's observed predictor value on that participant's outcome status (uptake vs. no uptake). Each participant had an estimated SHAP value for each predictor, and larger absolute SHAP values indicated a stronger effect of a predictor on the likelihood of a participant's uptake. We calculated each predictor's average absolute SHAP value across all participants in the training data. Additionally, we plotted SHAP values as a function of predictors to visualise the direction of the modelled relationship between the predictor and uptake. We used the bee swarm plotting method, in which participants' SHAP values for each predictor were plotted as a function of the predictor value. A positive relationship between a predictor and SHAP value indicated that higher values were related to a greater probability of uptake, and a negative relationship indicated the opposite. To supplement model interpretation, we also examined descriptive statistics for the predictors across participants who did versus did not achieve uptake in the full merged training and testing data, accompanied by *t*‐tests (for continuous variables) and chi‐square tests (for categorical variables) to denote which variables differed significantly across participants who did versus did not achieve uptake. SHAP values were estimated using the *shapper* (Maksymiuk et al. 2024) package in *R*.

## 3 Results

Table 2 displays performance in the training and testing data. The best model was SVM‐Linear, which achieved AUC of 0.70 and 70% accuracy in the testing data. LR performed similarly well, achieving 0.70 AUC and 64% accuracy in the testing data; the other two methods performed below acceptable standards for AUC, and neither model outperformed SVM‐Linear in terms of accuracy. There were no statistically significant differences between models in AUC based on the DeLong test or accuracy based on the McNemar test (see Table 3). Therefore, although there were no statistically significant differences in test set performance among the models, SVM‐Linear was one of only two models to achieve an acceptable AUC and also achieved the highest accuracy, and it was selected as the optimal model.

**TABLE 2:** Model performance.

Model	Training data				Testing data			
	Sensitivity (%)	Specificity (%)	Accuracy (%)	AUC	Sensitivity (%)	Specificity (%)	Accuracy (%)	AUC
LR	53	77	65	0.70	54	75	64	0.70
SVM‐linear	70	62	66	0.70	74	66	70	0.70
SVM‐polynomial	88	87	88	0.95	63	59	61	0.63
RF	61	54	58	0.57	66	62	64	0.64

*Note: N* = 234 in training data, *N* = 67 in testing data.

Abbreviations: AUC = area under the receiver operating characteristics curve, LR = logistic regression with elastic net regularisation, RF = random forest, SVM = support vector machine.

**TABLE 3:** Statistical comparison of model performance.

Reference model	Comparator model	DeLong test		McNemar test	
		*Z*	*p*	*x* ^2^ (1)	*p*
LR	SVM‐linear	0.55	0.580	0.90	0.343
LR	SVM‐polynomial	1.00	0.315	0.05	0.823
LR	RF	1.07	0.285	0.00	1.000
SVM‐linear	SVM‐polynomial	0.96	0.335	1.39	0.239
SVM‐linear	RF	0.90	0.367	0.41	0.522
SVM‐polynomial	RF	−0.06	0.948	0.06	0.814

*Note: N* = 234 in training data, *N* = 67 in testing data. Comparisons based on testing data performance. DeLong test evaluated difference in area under receiver operating characteristics curve. McNemar test evaluated difference in classification accuracy.

Abbreviations: LR = logistic regression with elastic net regularisation, RF = random forest, SVM = support vector machine.

**FIGURE 1:**
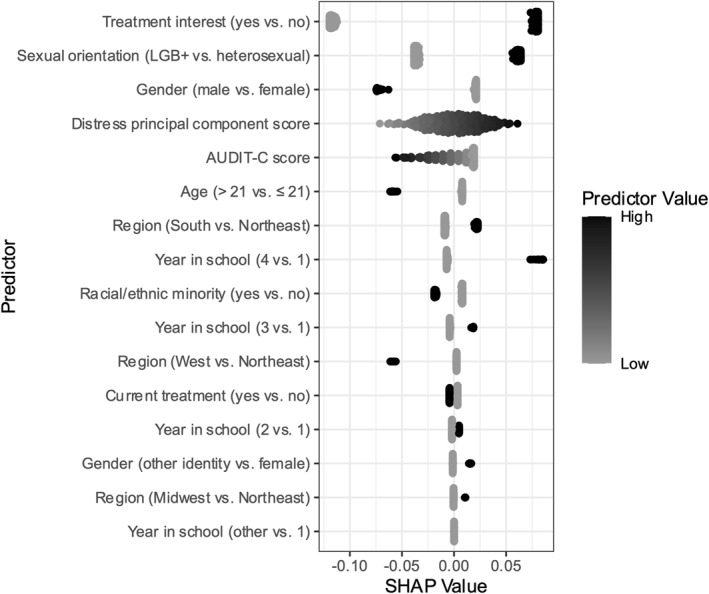
SHAP values as a function of predictor values. Points show individual participants from the training data (*N* = 234). Binary predictors are written such that the category after the ‘versus’ corresponds to the reference category (i.e., the lower of the two categories; thus, e.g., treatment interest = ‘yes’ corresponds to the higher value than treatment interest = ‘no’). Observed predictors were min‐max scaled prior to plotting for ease of comparison. A darker point denotes a higher scaled predictor value. A higher SHAP value indicates that a participant's observed score on a predictor contributed to a higher predicted probability of uptake. Thus, for a given predictor, if points become darker from the left of the graph to the right of the graph, higher values on that predictor corresponded to a higher predicted probability of uptake, and if points become lighter from the left of the graph to the right of the graph, higher values on that predictor corresponded to a lower predicted probability of uptake. AUDIT‐C = alcohol use disorders identification test, LGB+ = lesbian, gay, bisexual, or other sexual minority identity, SHAP = Shapley additive explanation.

Average absolute SHAP values from the selected model are shown in Table 4. The three strongest predictors of uptake were treatment interest (average absolute SHAP = 0.09), sexual orientation (average absolute SHAP = 0.05), and gender (male vs. female; average absolute SHAP = 0.03). For information about the nature of the relationships between predictors and uptake, please see SHAP values plotted in Figure 1 and descriptive characteristics of participants achieving versus not achieving uptake in Table 5. There were higher rates of uptake among participants reporting interest in treatment than those not reporting interest. Additionally, lesbian, gay, bisexual, and other sexual minority (LGB+) students achieved uptake at a higher rate than heterosexual students, and male students achieved uptake at a lower rate than female students. All three of these relationships were statistically significant in the full sample as indicated by chi‐square tests (see Table 5). SHAP values suggested that the distress principal component had a weak and positive relationship with uptake (i.e., more distressed students achieved higher uptake; average absolute SHAP = 0.02), and AUDIT‐C scores had a weak negative relationship with uptake (i.e., students with more problematic alcohol use achieved lower uptake; average absolute SHAP = 0.02), yet neither relationship was statistically significant in the full sample (see Table 5). No other variables demonstrated relationships with uptake. Thus, self‐reported treatment interest, gender, and sexual orientation were the most reliable predictors of uptake.

**TABLE 4:** Average absolute SHAP values.

Variable	Value
Treatment interest (yes vs. no)	0.09
Sexual orientation (LGB+ vs. heterosexual)	0.05
Gender (male vs. female)	0.03
Distress principal component score	0.02
AUDIT‐C score	0.02
Age (> 21 vs. ≤ 21)	0.01
Region (south vs. northeast)	0.01
Year in school (4 vs. 1)	0.01
Racial/ethnic minority (yes vs. no)	0.01
Year in school (3 vs. 1)	0.01
Region (west vs. northeast)	0.00
Current treatment (yes vs. no)	0.00
Year in school (2 vs. 1)	0.00
Gender (other identity vs. female)	0.00
Region (midwest vs. northeast)	0.00
Year in school (other vs. 1)	0.00

*Note:* SHAP values as estimated in training dataset (*N* = 234).

Abbreviations: AUDIT‐C = alcohol use disorders identification test, LGB+ = lesbian, gay, bisexual, or other sexual minority identity, SHAP = Shapley additive explanation.

**TABLE 5:** Predictors across uptake and non‐uptake participants.

Variable	No uptake (*N* = 143)		Uptake (*N* = 158)		Comparison	
	*N*	%	*N*	%	*x* ^2^ (*df*)	*p*
Treatment interest						
Yes	66	46	115	73	**21.11 (1)**	**< 0.001**
No	77	54	43	27		
Current treatment						
Yes	57	40	72	46	0.78 (1)	0.377
No	86	60	86	54		
Age						
18–21	122	85	143	91	1.46 (1)	0.227
22 or older	21	15	15	9		
Gender						
Female	95	66	121	77	**8.65 (2)**	**0.013**
Male	41	29	24	15		
Other identity	7	5	13	8		
Sexual orientation						
Heterosexual	104	73	84	53	**11.43 (1)**	**0.001**
LGB+	39	27	74	47		
Racial/ethnic minority status						
White, non‐hispanic	98	69	116	73	0.65 (1)	0.420
Racial/ethnic minority	45	31	42	27		
Year in school						
Undergraduate year 1	57	40	59	37	0.82 (4)	0.936
Undergraduate year 2	45	31	47	30		
Undergraduate year 3	26	18	31	20		
Undergraduate year 4	8	6	12	8		
Other	7	5	9	6		
School region						
Northeast	96	67	101	64	1.50 (3)	0.683
South	36	25	48	30		
Midwest	5	3	5	3		
West	6	4	4	3		

	*M*	SD	*M*	SD	*t* (299)	*p*
Distress principal component	−0.28	2.20	0.13	1.88	1.71	0.089
AUDIT‐C	2.67	2.63	2.36	2.36	−1.07	0.287

*Note:* Boldface denotes statistically significant difference between participants achieving vs. not achieving uptake.

Abbreviations: AUDIT‐C = alcohol use disorders identification test, LGB+ = lesbian, gay, bisexual, or other sexual minority identity.

## 4 Discussion

We aimed to develop and evaluate machine learning models to predict uptake into a self‐guided digital mental health intervention. The best‐performing model, a linear SVM model, achieved 70% accuracy and AUC of 0.70 in the test dataset. Despite achieving the highest accuracy and AUC, there were not statistically significant differences in performance between this model and the other models evaluated. The relatively small differences in performance of the models likely arose due to the modest sample size and commonalities across the models (e.g., making use of the same training data). The linear SVM model still achieved acceptable performance in absolute terms in addition to its modest and non‐significant relative advantages over the other models evaluated, and therefore it emerged as the optimal model for predicting uptake. The variable most strongly related to uptake was a single‐item self‐report question about interest in receiving mental health services. Being LGB+ (vs. heterosexual) was associated with a higher probability of uptake, and being male (vs. female) was associated with a lower probability of uptake. The results suggest that machine learning methods hold promise in predicting uptake to online mental health programs and shed light on factors associated with uptake.

The best‐performing model achieved an AUC of 0.70, which is considered acceptable (Hosmer et al. 2013). The AUC of the best model in the present study was substantially higher than models reported by Linardon et al. (2022; 0.50) and slightly higher than the training dataset performance reported by Cross et al. (2022; 0.66). Vera Cruz et al. (2023) reported marginally higher accuracy (71% vs. 70% in the present study), although their modelling problem differed from the present study's given that it concerned self‐reported use of digital health programs in a general adult sample (as opposed to objective uptake among persons referred for a specific program, as in the present study). The present study analyses are especially relevant to program triage and referral given that they examined uptake immediately after symptomatic participants were provided access to specific programs. Thus, the results presented here suggest an improvement from prior efforts to predict objective online intervention uptake using machine learning.

Analyses of substantive model implications suggested that the strongest predictor of uptake was self‐reported interest in receiving mental health treatment. Although this finding is intuitive, it is novel and has implications for personalisation of mental health care. Previous machine learning investigations (e.g., Cross et al. 2022), in addition to the present study, have used a range of demographic and clinical characteristics in algorithms predicting uptake. Outside of clinical trial settings, such a wide range of background characteristics might not be available prior to allocation of an intervention, and there might be limited time for assessment. Given that interest in treatment was assessed with a single‐item and was so predictive of uptake, it could prove useful and easy to include this item in other settings in which predicting uptake is of interest. For example, given evidence about effectiveness of motivational interviewing (Dean et al. 2016) and text message‐based reminders (Fitzsimmons‐Craft et al. 2024) as methods of increasing mental health service uptake, these forms of supplementary intervention could be administered to distressed persons not reporting interest in treatment to increase the probability of uptake. The exact manner of assessing treatment interest would naturally vary across contexts. As one possibility, a digital self‐help provider could use a customer service chatbot to ask about interest in services on its public website. Based on responses, the chatbot might subsequently refer those reporting interest to available services while using motivational interviewing methods to increase interest in services among those not reporting interest. We recommend assessing interest in treatment where possible in other settings where predicting uptake is of interest.

The model also identified gender as a predictor of uptake, with female participants achieving higher uptake than males. This gender difference corresponds with the findings of Vera Cruz et al. (2023), who also studied a U.S. sample, yet it differs from the findings of Cross et al. (2022), who found higher uptake in males (vs. females) in an Australian sample. In the U.S., cultural masculinity norms have been associated with mental health stigma and less positive attitudes about mental health services among men (Vogel et al. 2011). In Australia, public help‐seeking and stigma reduction initiatives have reduced gaps in mental health service use among men with mental disorders (Harris et al. 2015), potentially explaining differential associations between gender and digital mental health service uptake in these countries. Thus, especially in the U.S., efforts should be taken to alleviate the impact of stigma on service uptake among men. Of note, U.S. men are not the only segment of the population at risk of experiencing mental health stigma. Therefore, irrespective of setting, providers of digital mental health services should take special care to ensure that stigma does not impede uptake in the populations they are trying to reach. Meta‐analytic evidence has suggested that educational interventions (e.g., providing information about the prevalence of mental health concerns and the benefits of seeking help) can reduce stigma and can be effectively administered online (Griffiths et al. 2014). Thus, uptake might be improved among U.S. men and other populations at risk of experiencing stigma through effective educational resources provided alongside digital health service referrals.

No prior machine learning analyses have examined sexual orientation as a predictor of online intervention uptake, though we found that LGB+ participants were more likely to achieve uptake than heterosexual participants. The present study's finding converges with recent evidence that, among U.S. college students with probable mental health problems, LGB+ students were more likely to receive psychotherapy (Rackoff et al. 2025). Of note, another study documenting greater psychotherapy utilization among LGB+ students indicated that some LGB+ students were also less likely to seek support from other sources such as family or clergy (Baams et al. 2018). Plausibly, for some LGB+ students, professional mental health services are less associated with concerns about non‐acceptance than other sources of social support, leading them to achieve uptake at higher rates. Indeed, epidemiological research has indicated that receiving social support from friends or family is associated with reduced odds of using speciality mental health services (Maulik et al. 2011), suggesting that services might be used at greater rates by those with insufficient support in their social networks. Sources of social support were not assessed in the present study, though they would be important to assess in future research both as possible mediators of LGB+ students' greater uptake and as possible features to improve predictive accuracy.

Several of the putative predictor variables were not found to be related to uptake. Alcohol abuse did not have a substantial relationship with uptake, differing from two prior studies (Cross et al. 2022; Vera Cruz et al. 2023). Furthermore, whereas prior literature yielded mixed findings on the relationship between age or mental health symptom severity and online program uptake (Cross et al. 2022; Vera Cruz et al. 2023), neither variable predicted uptake in the present study. The heterogeneity of results across studies suggests that some variables' relationships with uptake depends on the population and treatment setting. Therefore, in practice, machine learning models used to predict uptake should be built and evaluated in settings similar to those in which they will be deployed. Doing so is likely to maximise the models' accuracy and utility.

Several limitations of the present study deserve note. First, the sample size was relatively small (*N* = 301 across training and testing datasets). This might have limited models' abilities to detect weak but real relationships between certain predictors and uptake, including nonlinear and interactive relationships. Additionally, the sample consisted of U.S. college students, and therefore the model performance and predictive relationships observed might differ from other samples (e.g., non‐U.S. samples or samples with different age ranges or occupational statuses). Finally, this study was not pre‐registered, and gold standards for model evaluation suggest prospective evaluation of model performance in a completely new dataset (e.g., Brajer et al. 2020), yet collecting such data was not feasible for the present study. Notwithstanding these limitations, the present findings illustrate the potential for machine learning methods to identify probable users of online interventions.

Uptake is a critical bottleneck to the impact of online mental health interventions. The present study aimed to build and evaluate machine learning models to predict who would achieve uptake using baseline survey data from a randomised trial. The best‐performing model achieved an accuracy of 70% and AUC of 0.70 in a testing dataset, reflecting acceptable performance. Self‐reported mental health treatment interest, sexual orientation, and gender were the most reliable predictors of uptake. It is possible that machine learning methods could be applied in future triage settings to identify persons who might benefit from motivational enhancement to engage with self‐guided online interventions. Additionally, it would be valuable to understand factors underlying male and heterosexual students' lower probability of uptake. Such efforts may inform improvements in interventions and their methods of delivery, maximising real‐world impact.

## Author Contributions

Gavin N. Rackoff formulated the research question, analysed the data, and drafted the manuscript under Michelle G. Newman's supervision. Michelle G. Newman provided critical revisions.

## Ethics Statement

All research procedures were reviewed and approved by the Institutional Review Board of The Pennsylvania State University.

## Conflicts of Interest

The authors declare no conflicts of interest.

## Supporting information

Supporting Information S1

## Data Availability

The data that support the findings of this study are openly available in Open Science Framework at https://osf.io/y7t54/?view_only=bb1d3277adff4572b6313636b29f14a2.

## Endnotes

^1^
We selected six as the number of folds for cross‐validation because the training sample was evenly divisible by six, leading to equally sized folds and simplifying computations in manually coded grid search model tuning.
