# Supplementary material for: Using Machine Learning to Predict Uptake to an Online Self‐Guided Intervention for Stress During the COVID‐19 Pandemic
Source: Stress Health. 2025 Apr 22;41(2):e70032. doi: 10.1002/smi.70032 (PMC12013697; doi:10.1002/smi.70032)
Supplement: Supplementary file 1 — Supporting Information S1 [file SMI-41-e70032-s001.docx]

***Intervention Descriptions Shown to Participants***

Please see Supplementary Figure 1 for the description of the intervention shown to participants in the Qualtrics survey and Supplementary Figure 2 for the email sent to participants who did not achieve uptake immediately.

***Train-Test Partition***

To ensure that no demographic groups were over- or under-represented in the training dataset, stratified sampling was used to randomly select approximately three fourths of the dataset for training while balancing the following variables across the train and test datasets: school region, school sector (private vs. public), gender identity, sexual orientation, racial minority status. This procedure resulted in 234 (78%) participants being selected to the training dataset, with the remaining 67 (22%) participants reserved for the testing dataset. Given that the dependent variable, uptake, occurred at close to a 50% rate sample-wide, it was unnecessary to stratify by this variable. The resulting training and testing datasets therefore had similar composition across the demographic variables used for stratified sampling and uptake (see Supplementary Table 1).

***Data Preparation***

**Data Cleaning.** Given the presence of age outliers (i.e., students whose ages were well outside the typical college age range of 18-22) and the strong overlap between age and year in school among students within the typical college age range, age was categorized as a two-group variable: 18 to 22 years old vs. over 22 years old. This mitigated possible ill effects of outliers and multicollinearity on model performance. Additionally, minor missing data was present on several of the predictor variables (GAD-Q-IV, ISI, PC-PTSD, AUDIT-C). Therefore, linear regression imputation was implemented by fitting a regression model in the training data in which all predictors with complete data were modeled as predictors of the variable with missing data, and the resulting regression equation was used to estimate and impute probable scores on each missing predictor for any participant with missing data. The same regression equation (as fit on the training data) was implemented to estimate and impute missing values in the testing data. Of note, there was no missing data on the outcome variable, uptake, and therefore this method of missing data handling does not affect the validity of predictive performance estimates. Visual inspection in the training data also suggested no relationship between school sector and uptake, and this variable was therefore not retained as a predictor in machine learning models.

**Dimension Reduction.** The large number of mental health symptom predictors posed the potential for collinearity among predictors, which could worsen model performance. We therefore performed principal components analysis (PCA) to reduce the 11 mental health symptoms to a smaller number of predictors. PCA was performed on standardized scores of the mental health symptom variables in the training dataset, and principal component scores were subsequently calculated in both the training and testing datasets by multiplying principal component loadings by standardized scale scores. Inspection of the principal components as estimated in the training data revealed that the first principal component explained a substantial proportion of variance across the symptom predictors (38%), and subsequent principal components explained a much smaller proportion of variance (10% or less). The first principal component had positive standardized loadings from all mental health predictors except AUDIT-C, and the predictors with the strongest loadings were PHQ-9, PSS-10, and DASS-Depression (see Supplementary Table 2). Thus, we considered the first principal component to be an indicator of general distress, which captured variance in the majority of the symptom measures. Given that the AUDIT-C was not well captured by this principal component, we retained both the first principal component and AUDIT-C scores as predictors in model building, alongside the other demographic, school-related, and treatment seeking-related predictors assessed in the survey. Omitting the PCA step from analysis did not yield a better-performing model in the testing data than the best-performing model of those trained using dimension-reduced data (see Supplementary Table 3).

***Modeling Strategies***

Logistic regression estimates linear associations between the predictors and the log odds of the dependent variable. SVM-Linear and SVM-Polynomial both identify a hyperplane across all predictors that optimally classifies observations on the dependent variable. For SVM-Polynomial, the polynomial kernel function allows for nonlinear decision boundaries along continuous predictors (i.e., general distress and AUDIT-C), whereas for SVM-Linear, all decision boundaries are linear. RF aggregates decisions across a set of classification trees fit with bootstrap resampling and a limited subset of predictors per split in the classification tree. Each decision tree is built with recursive splits, enabling RF to capture interactive and non-linear associations between predictors and the dependent variable.

Tuning was performed using 6-fold cross validation performed in the training data. That is, the training data were split into 6 random folds, a model with specified tuning parameter(s) was fit to a dataset consisting of all but one fold, and the performance of the model was evaluated by examining its predictive accuracy on the held-out fold. This procedure was conducted with each fold serving as a hold-out fold, and the best performing set of tuning parameters was the set for which prediction accuracy was highest on average across all 6 hold-out folds. Tuning grids are in Supplementary Table 4.

***Program Selection***

Across the entire sample, among those who achieved uptake (*N* = 158), there were no significant differences on the predictor variables among participants who initially selected the positive psychology program, participants who initially selected the COVID-19 coping program, and participants who did not select an initial program after accessing the intervention platform. Descriptive characteristics and statistical comparisons (with Fisher’s exact test for categorical variables and ANOVA for continuous variables) are shown in Supplementary Table 5.

| Supplementary Table 1 |  |  |
| --- | --- | --- |
| *Stratification Results* |  |  |
| Variable | Train *N* | Test *N* |
| Uptake |  |  |
| Yes | 123 | 32 |
| No | 111 | 35 |
| Gender |  |  |
| Female | 165 | 51 |
| Male | 52 | 13 |
| Other identity | 17 | 3 |
| Sexual Orientation |  |  |
| Heterosexual | 146 | 42 |
| LGB+ | 88 | 25 |
| Racial minority status |  |  |
| White, non-Hispanic | 164 | 50 |
| Minority or not disclosed | 70 | 17 |
| Region |  |  |
| Midwest | 9 | 1 |
| Northeast | 148 | 49 |
| South | 68 | 16 |
| West | 9 | 1 |
| Sector |  |  |
| Private | 18 | 1 |
| Public | 216 | 66 |

| Supplementary Table 2 | |
| --- | --- |
| *Standardized Loadings for First Principal Component* | |
| Variable | Loading |
| PHQ-9 | 0.41 |
| PSS-10 | 0.36 |
| DASS-Depression | 0.35 |
| DASS-Anxiety | 0.33 |
| ISI | 0.32 |
| SPDQ | 0.31 |
| GAD-Q-IV | 0.31 |
| DASS-Stress | 0.31 |
| PTSD | 0.25 |
| WCS | 0.14 |
| AUDIT-C | -0.04 |

| Supplementary Table 3 | | | | | | | | | |
| --- | --- | --- | --- | --- | --- | --- | --- | --- | --- |
| *Model Performance in Models Without Principal Components Analysis* | | | | | | | | | |
| Model | Training Data | | | |  | Testing Data | | | |
|  | Sensitivity | Specificity | Accuracy | AUC |  | Sensitivity | Specificity | Accuracy | AUC |
| LR | 54% | 77% | 65% | .70 |  | 54% | 72% | 63% | .70 |
| SVM-Linear | 78% | 56% | 68% | .71 |  | 74% | 56% | 66% | .69 |
| SVM-Polynomial | 78% | 56% | 68% | .71 |  | 74% | 56% | 66% | .69 |
| RF | 72% | 37% | 55% | .52 |  | 69% | 34% | 52% | .60 |

*Note*. *N* = 234 in training data, *N* = 67 in testing data. LR = logistic regression with elastic net regularization. SVM = support vector machine. RF = random forest. AUC = Area under the receiver operating characteristics curve. Parameters selected with 6-fold cross-validation were LR Alpha = 0.1, LR Lambda = 0.3, SVM-Linear cost = 0.01, SVM-Polynomial cost = 10, SVM-Polynomial degree = 1, SVM-Polynomial Gamma = 0, RF number of variables = 15 (note: to accommodate more variables given the absence of dimension reduction, we included the values of 3, 6, 9, 12, and 15 in the grid instead of the original values of 2, 4, 6, 8, and 10), RF number of trees = 50. The selected thresholds were .55 (logistic regression), .50 (linear support vector machine), .50 (polynomial support vector machine), and .44 (random forest).

| Supplementary Table 4 |  |  |
| --- | --- | --- |
| *Tuning Grids and Selected Parameter Values for Models Reported in Main Manuscript* | | |
| Tuning Parameter | Evaluated Values | Selected Value |
| LR |  |  |
| Alpha | 0.0, 0.1, 0.2, 0.3, 0.4, 0.5, 0.6, 0.7, 0.8, 0.9, 1.0 | 0.1 |
| Lambda | 0.0, 0.1, 0.2, 0.3, 0.4, 0.5, 0.6, 0.7, 0.8, 0.9, 1.0 | 0.3 |
| SVM-Linear |  |  |
| Cost | 0.001, 0.005, 0.01. 0.05, 0.1, 0.5, 1, 5, 10, 50 | 0.01 |
| SVM-Polynomial |  |  |
| Cost | 0.001, 0.005, 0.01. 0.05, 0.1, 0.5, 1, 5, 10, 50 | 5 |
| Degree | 1, 2, 3, 4 | 3 |
| Gamma | 0.001, 0.005, 0.01. 0.05, 0.1, 0.5, 1, 5, 10, 50 | 0.05 |
| Random Forests |  |  |
| Number of variables | 2, 4, 6, 8, 10 | 8 |
| Number of trees | 10, 50, 100, 500, 1,000 | 100 |

*Note*. *N* = 234 in training data. After hyperparameter tuning, we also selected the threshold value that maximized the Youden index in the training data, evaluating across all possible thresholds between 0 and 1. The selected thresholds were .56 (logistic regression), .55 (linear support vector machine), .56 (polynomial support vector machine), and .52 (random forest).

| Supplementary Table 5 |  |  |  |  |  |  |  |  |
| --- | --- | --- | --- | --- | --- | --- | --- | --- |
| *Predictor Characteristics Across Program Selection Groups* | | |  |  |  |  |  |  |
| Characteristic | Selected Program | | | | | | Statistical Comparison | |
|  | General Resilience | | COVID-19 Stress | | No Selection | |  |  |
|  | *M* | *SD* | *M* | *SD* | *M* | *SD* | ANOVA | |
| Distress Principal Component | 0.12 | 1.79 | 0.77 | 1.64 | -0.67 | 2.55 | *F*(2,155) = 2.51, *p* = .085 | |
| AUDIT-C Score | 2.44 | 2.33 | 1.68 | 2.63 | 2.6 | 2.26 | *F*(2,155) = 0.93, *p* = .397 | |
|  | *N* | % | *N* | % | *N* | % | Fisher's Exact Test | |
| Age > 21 (vs. ≤ 21) | 13 | 10 | *2* | 11 | 0 | 0 | *p* = .547 | |
| Treatment Interest = yes (vs. no) | 90 | 73 | 15 | 79 | 10 | 67 | *p* = .731 | |
| Current Treatment = yes (vs. no) | 56 | 45 | 9 | 47 | 7 | 47 | *p* > .999 | |
| Racial/Ethnic Minority = yes (vs. no) | 34 | 27 | 5 | 26 | 3 | 20 | *p* = .945 | |
| Sexual Orientation = LGB+ (vs. heterosexual) | 60 | 48 | 9 | 47 | 5 | 33 | *p* = .608 | |
| Gender = Female | 97 | 78 | 14 | 74 | 10 | 67 | *p* = .274 | |
| Gender = Male | 16 | 13 | 3 | 16 | 5 | 33 |  |  |
| Gender = Other gender identity | 11 | 9 | 2 | 11 | 0 | 0 |  |  |
| Year in school = 1 | 49 | 40 | 4 | 21 | 6 | 40 | *p* = .612 | |
| Year in school = 2 | 38 | 31 | 4 | 21 | 5 | 33 |  |  |
| Year in school = 3 | 23 | 19 | 4 | 21 | 4 | 27 |  |  |
| Year in school = 4 | 8 | 6 | 4 | 21 | 0 | 0 |  |  |
| Year in school = Other | 6 | 5 | 3 | 16 | 0 | 0 |  |  |
| Region = Northeast | 77 | 62 | 12 | 63 | 12 | 80 | *p* = .880 | |
| Region = Midwest | 4 | 3 | 1 | 5 | 0 | 0 |  |  |
| Region = South | 39 | 31 | 6 | 32 | 3 | 20 |  |  |
| Region = West | 4 | 3 | 0 | 0 | 0 | 0 |  |  |

**Supplementary Figure 1:** Intervention description provided in survey


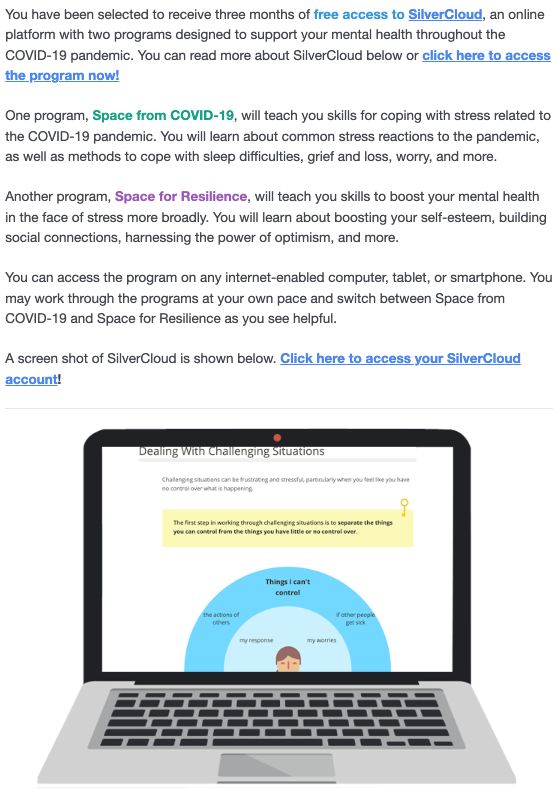


**Supplementary Figure 2.** Email reminder to access the program.

Hi [participant],

This is [study team member] from the COVID-19 Mental Health Study. Thank you very much for participating in the baseline survey! I really appreciate you taking the time to help us understand college student mental health during the pandemic.

As a reminder, you were randomly selected to receive access to **SilverCloud**, an online platform with two programs designed to support your mental health during the pandemic. **You can create an account and begin using the program by following the link below:**

[link]

Please keep an eye out for the one-month follow-up survey on [date]. You would be placed into a raffle for a $25 Amazon gift card for completing the follow-up survey.

Kind regards,

[study team member] from the study team
